# Inflammation Amplification by Versican: The First Mediator

**DOI:** 10.3390/ijms13066873

**Published:** 2012-06-06

**Authors:** Zhenwei Zhang, Lei Miao, Lianghua Wang

**Affiliations:** 1 Department of Biochemistry and Molecular Biology, Second Military Medical University, Shanghai 200433, China; E-Mail: zhengwei-523@hotmail.com; 2 Department of Pharmacology, Zhejiang Chinese Medical University, Hangzhou 310053, China; E-Mail: miaoleimolly@126.com

**Keywords:** versican, cytokines, extracellular matrix homeostasis, inflammation amplification

## Abstract

The effects of inflammation may not always benefit the individual. Its amplifying nature represents a highly regulated biological program, and the inflammatory microenvironment is its essential component. Growing evidence suggests that the ECM (extracellular matrix) is important for the early steps of inflammation. Versican, a ubiquitous component of the ECM, contributes to the formation of the inflammatory response and is highly regulated by cytokines. Certain cytokines exert their initial effects on versican to alter the homeostasis of the inflammatory milieu, and inappropriate production of versican may promote the next inflammatory response. Therefore, versican could be the first step in the amplification of the inflammatory response, and ongoing research of this molecule may help to explain the pathogenesis of inflammation.

## 1. Introduction

Although inflammation helps to fight infection, it often results in an exacerbation of a diseased state in the individual [1]. The concept of inflammation as a feature of cancer is now generally accepted [2,3]. It has been suggested that “smoldering” inflammation comprises a diverse range of processes that affect every aspect of pathology and even normal physiology. In tumorigenesis, inflammation acts at all stages, and, in chronic, progressive disorders, uncontrolled inflammation drives the chronic progression of neurodegenerative diseases [4,5]. Moreover, the inflammatory response, which has an amplifying nature, represents a highly regulated biological program, and the inflammatory microenvironment is its essential component [4–6].

The extracellular matrix (ECM), which is mainly composed of proteoglycans, glycoproteins and collagens that surround and support the cells within tissues, is a complex structural entity with many physiological and pathological roles. The ECM maintains tissue integrity and homeostasis and provides a reservoir of cytokines and growth factors. However, a number of reports have suggested that the ECM is much more than a scaffold or a buffering environment for ions and neurotransmitters. Instead, it has been proposed that the three-dimensional ultrastructure and its individual components exert a profound effect on cells and modulate the basic functions that are important for the early steps in inflammation, such as the immune cell migration into inflamed tissues and immune cell differentiation [7]. Aberrant ECM contains proteoglycans and glycoproteins in higher proportions than in the normal surrounding tissue [8] and may create a more favorable, suitable and hospitable extracellular environment for inflammation; such a situation influences immune cell activation and survival and contributes to immune responses [7]. Furthermore, numerous extracellular signaling proteins, such as chemokines, cytokines and growth factors, are associated with and exhibit activity in the inflamed ECM, suggesting a possible link between the ECM and inflammation. However, as the relevant data are limited and fragmentary, it is unclear whether the remodeled ECM of inflamed tissues affects the propagation of the inflammatory response [7]. Accordingly, it is not surprising that the study of the remodeling of the ECM that contributes to inflammation has become a novel research topic.

## 2. Versican and Its Structure

**Figure 1:**
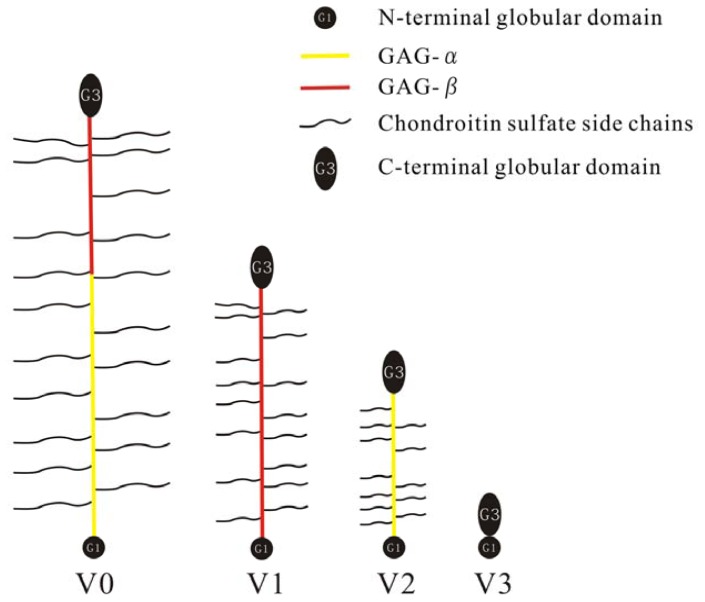
Schematic diagram of versican isoforms.

Versican, a ubiquitous component of the ECM, is highly expressed by proliferating cells and mesenchyme during tissue remodeling and embryonic morphogenesis [9–11]. Structurally, versican consists of an *N*-terminal globular domain (G1), a *C*-terminal globular domain (G3), and chondroitin sulfate (CS) chain-binding regions (GAG-α, GAG-β) between the G1 and G3 domains [12]. Alternative splicing generates at least four isoforms of versican, known as V0, V1, V2, and V3 [13] (Figure 1). V0 contains both GAG-α and GAG-β; V1 and V2 possess only GAG-β and GAG-α, respectively; and V3 solely has the globular domains. Versican isoforms V0/V1 are mainly expressed in the late stage of embryonic development [14], whereas versican V2 is one of the main constituents of the mature CNS ECM [15].

The G1 region of versican is composed of an immunoglobulin fold followed by a contiguous pair of link modules that bind hyaluronan; the G3 region contains two epidermal growth factor (EGF)-like repeats, a carbohydrate recognition domain (a lectin-like repeat), and a complement regulatory protein-like motif [16]. Therefore, versican has diverse binding partners through its different molecular motifs. For example, versican binds to hyaluronan through its G1 domain, to CD44 and L and P selectins through its CS chains, and to epidermal growth factor receptor (EGFR), tenascin-R, fibulin-1 and -2 through its *C*-terminal domain (G3) [12]. In addition, versican binds to P selectin glycoprotein ligand-1 and has a role in mediating leukocyte aggregation [17]; it also binds to adhesion molecules on the surface of inflammatory leukocytes. In particular, because versican binds to both hyaluronan and CD44, it is possible that the three may form complexes together and stabilize the CD44-dependent interactions of inflammatory cells.

## 3. Cytokines Exert Their Effects on Versican

Given that several cytokines are critical for both inflammation and tumor growth, they control the hubs of protumorigenic signaling and may be targeted to curtail both tumor-associated inflammation and tumor growth [4]. In most cases, cytokines or growth factors act in an autocrine or a paracrine manner to control the immune and inflammatory milieu [18]; these molecules exert their functions in the inflammatory microenvironment and function as important mediators of harmful inflammation, either to favor antitumor immunity or enhance tumor progression [19]. For instance, the perpetuation of chronic inflammation, which is largely achieved through positive feedback loops, may depend mostly on the cytokines of the consecutive autocrine or paracrine system and the prolonged recruitment of inflammatory cells into the inflammatory milieu. Certain cytokines, such as IL-1β, TNF-α, and interferon-γ, have received much attention with regard to the neuroinflammatory processes in neurodegeneration [20].

As demonstrated by its unique structural features, versican creates a highly interactive molecular environment and binds to a variety of ECM components, cytokines and growth factors to influence the fundamental events involved in the inflammatory response. It has been shown that the synthesis of versican is highly regulated by specific cytokines (Table 1). Indeed, numerous studies have reported that specific cytokines or growth factors, such as transforming growth factor β1, β2 and β3 (TGF-β1, -β2 and -β3), basic fibroblast growth factor (bFGF), platelet-derived growth factor BB (PDGF-BB), and Interleukin-1β (IL-1β), regulate the synthesis of versican [21–25]: IL-1β down-regulates the expression of versican by arterial smooth muscle cells, TGF-β1 up-regulates versican in prostatic fibroblasts, facilitating invasion of cancer cells [21,24], and TGF-β3 regulates the versican variant expression in conjunction with the disrupted ECM in leiomyogenesis [23]. Furthermore, two pro-inflammatory cytokines, TNF-α and IFN-γ, enhanced the transcription of versican V2 in neural precursor cells in a dose-dependent manner [26], while IFN-γ reduces versican expression in bleomycin-exposed lung fibroblasts [27]. In addition, it was demonstrated in our recent research that IL-11 could regulate the expression of versican and its variants in gastric cell lines, and versican, together with IL-11, participate in the regulation of gastric cell migration [28]. Findings in our experiments previously hinted at the answer: some neglected cytokines may join in the versican and even other extracellular matrix proteoglycan regulation. Thus, the extensive, and still growing, body of evidence discussed above indicates that versican and specific cytokines are probably involved together in the steps of inflammation.

## 4. Versican and Inflammation

In the last decade, research has elucidated a significant involvement of versican in tumors and neurodegenerative diseases; these observations, coupled with those demonstrating that versican regulates many of the events that contribute to the poor prognosis of cancer and neurodegeneration, highlight the critical importance of versican in the pathogenesis of disease [29–35]. It has been suggested that abnormal expression of versican is related to cell proliferation, differentiation, adhesion, and even the homeostasis and integrity of the ECM [36].

There is also evidence showing that versican mediates cell-ECM interaction and that the stable expression of versican enhances cell attachment and the expression of β1 integrin and fibronectin [37]. In addition, an *in vivo* study reveals that versican could stimulate mesenchymal to epithelial transition (MET) of metastatic tumor cells by attenuating phospho-Smad 2 levels, which result in elevated cell proliferation and accelerated metastases [38]. And an *in vitro* study shows that both versican and hyaluronan support macrophage adhesion [39]. Stimulation of mononuclear cells with granulocyte/macrophage colony-stimulating factor (GM-CSF) in isolated human peripheral blood mononuclear cells increases the expression of versican as well as cytokine induction, and the upregulation of versican during myocardial infarction has a role in the inflammatory reaction, which mediates the subsequent differentiation of monocytes in the infarcted heart [40]. In UVB-treated Ogg1 knockout mouse (lacking the repair enzyme 8-oxoguanine glycosylase), high versican expression is accompanied by inflammatory response, particularly neutrophil infiltration [41]. It is proved that polyinosine-polycytidylic acid stimulates versican accumulation in the ECM, and the accumulation could promote monocyte adhesion [42]. An adenine to thymine transversion at the highly conserved splice acceptor site in intron 7 of the versican gene which produces aberrantly spliced versican transcripts in a French Wagner family is associated with vascular and inflammatory ocular features [43]. Notably, versican has been suggested to interact with a small number of metastatic cells and activate macrophages through toll-like receptor 2 (TLR2) and its co-receptors, TLR6 and CD14, leading to the production of the metastasis promoting cytokine, TNF-α, in addition to macrophage activation [44]. Ligation of TLR2 by versican appears to be directly involved in the activation of multiple types of cells in tumor stroma and the induction of inflammatory cytokine secretion [45]. Such interactions may sustain the inflammatory response, and could thus place versican at the top of the list of molecules to target in attempts to control inflammation [36]. By localizing TGF-β in the ECM and regulating its signaling, versican could facilitate chondrogenesis and joint morphogenesis [10] (Table 2). Taken together, these results led the authors to envision that the ECM component contributes to the formation of an inflammatory response.

## 5. Versican Could Be the First Step in the Amplification of the Inflammatory Response

It is pointed out that versican and other extracellular-matrix proteoglycans collectively serve as a repository, containing essential cytokines that stimulate ECM remodeling via the autocrine and paracrine mechanisms [46,47]. Through altering the levels of versican and/or hyaluronan, cytokines stimulate the formation of versican-hyaluronan aggregates and contribute to the remodeling of the ECM [48,49]. So, to elicit an effective immune response, some cytokines that serve as mediators or amplifiers of the program of inflammation may exert their initial effects on the extracellular matrix to change the homeostasis of the inflammatory milieu by regulating particular ECM components, such as versican. This altered homeostasis creates a favorable environment for the cytokines and growth factors and also promotes the recruitment of new inflammatory cells, such as macrophages and neutrophils. Some interesting studies have also demonstrated that versican is a TLR2 agonist and metastasis-enhancing factor and that it can interact with inflammatory chemokines and partially regulate their activity [44,50,51]. Additionally, a highly inflammatory microenvironment could induce high versican expression, with high numbers of infiltrated neutrophils. Conversely, neutrophil infiltration induces versican overexpression [41]. Ultimately, the ‘cytokine storm’ arrives, causing uncontrolled, prolonged inflammation. A vicious circle between the aberrantly expressed versican and the uncontrolled inflammatory cytokines inevitably occurs and further contributes to the ongoing pathology.

Within this context, versican appears to play a more significant role than merely contributing to the impaired homeostasis in the initially hostile microenvironment; it also mediates the dialog between inflammation and the cytokines. Hence, we propose that versican could be the first step in the amplification of inflammation.

## 6. Concluding Remarks

Although the amplifying nature of inflammation has yet to be fully elucidated, interpreting the basis for ECM and its components in the inflammatory processes is important in this endeavor. Given their important roles, versican and certain cytokines may become promising targets for the development of therapeutic agents that halt the cycle of the inflammatory response and, thereby, retard the progression of inflammation. Taken together, this research adds a new dimension to our understanding of the process of inflammation by demonstrating that versican is required in its amplification. Thus, regardless of the origin of the inflammatory process, therapeutic intervention aimed at the prevention or downregulation of versican could be of great use in ameliorating the amplifying nature of the progression of inflammation and even halt the inflammatory response. Therefore, future directions will be to develop molecular genetic models that reflect the diversity of inflammation and prevent or interrupt the vicious circle between dilapidated interstitial matrix and orderless cytokines so as to retard the progression of inflammation, for from such work innovative therapeutic strategies may follow.

## Figures and Tables

**Table 1:** Summary of cytokine effects on versican.

Cytokine	Effects	References
TGF-β1	TGF-β1 up-regulates versican mRNA levels and core protein synthesis in prostatic fibroblasts, facilitating invasion of cancer cells.	[21,27]
TGF-β2	TGF-β2 triggers a significant increase of versican V0 and V1 mRNAs and proteins in some fibrosarcoma cells and osteosarcoma cells.	[22,28]
TGF-β3	TGF-β3 up-regulates the versican variant expression in conjunction with the disrupted ECM in leiomyogenesis.	[23]
bFGF	Compared to TGF-β2, bFGF elucidates minor stimulatory effects on versican and its isoforms expression in osteosarcoma cells.	[22]
PDGF-BB	PDGF-BB stimulates versican core protein expression in monkey arterial smooth muscle cells.	[25]
IL-1β	IL-1β selectively down-regulates versican synthesis by arterial smooth muscle cells, while positively regulating the synthesis of other protroglycans.	[24]
IL-11	IL-11 could up-regulate the expression of versican and its variants in gastric cell lines.	[28]
TNF-α	TNF-α enhances the transcription of versican V2 in neural precursor cells in a dose-dependent manner.	[26]
IFN-γ	IFN-γ reduces versican expression in bleomycin-exposed lung fibroblasts, While exerting the same effects on versican V2 as TNF-α in neural precursor cells.	[26,27]

**Table 2:** Role of versican in inflammation-associated facts.

Fact	Effects	References
Aberrant homeostasis	The abnormal expression of versican is related to the homeostasis and integrity of the ECM.	[36]
	Versican could stimulate mesenchymal to epithelial transition.	[38]

Inflammatory cell recruited and activated	Versican supports and activates macrophage adhesion.	[39,44]
	High versican expression is accompanied by neutrophil infiltration.	[41]
	The accumulation of versican in the ECM could promote monocyte adhesion.	[42]
	The upregulation of versican mediates the differentiation of monocytes in the infarcted heart.	[40]

TLR signal activated	Versican activates TLR2 and its co-receptors, TLR6 and CD14.	[44]

Cytokines localized and induced	Versican could localize TGF-β in the ECM and regulating its signaling, facilitating chondrogenesis and joint morphogenesis.	[10]
	Versican induces the production of TNF-α.	[44]
